# Ethical and social implications of approaching death prediction in humans - when the biology of ageing meets existential issues

**DOI:** 10.1186/s12910-020-00502-5

**Published:** 2020-07-27

**Authors:** Marie Gaille, Marco Araneda, Clément Dubost, Clémence Guillermain, Sarah Kaakai, Elise Ricadat, Nicolas Todd, Michael Rera

**Affiliations:** 1 Université de Paris, SPHERE, UMR 7219, CNRS-Université Paris Diderot, bâtiment Condorcet, case 7093, 5 rue Thomas Mann, 75205 Paris, France; 2 Université de Paris, CRPMS - EA 3522, IUH - EA 3518, bâtiment Olympe de Gouges, 8 rue Albert Einstein, 75013 Paris, France; 3 Head of intensive care unit, Begin military hospital & CognacG research unit, UMR CNRS-Paris Descartes-SSA, Paris, France; 4 Laboratoire Manceau de Mathématiques, Institut du Risque et de l’Assurance, Le Mans Université, 72000 Le Mans, France; 5 Max Planck Institute for Demographic Research, Rostock, Germany; 6 Center for Research and Interdisciplinarity (CRI), Université de Paris, INSERM U1284. Sorbonne Université, IBPS, B2A, CNRS, Institut de Biologie Paris – Seine, 75005 Paris, France

**Keywords:** Ageing, Anticipation, Death, Longevity risk, Medical care, Mortality, Prediction, Pre-symptomatic test, Program

## Abstract

**Background:**

The discovery of biomarkers of ageing has led to the development of predictors of impending natural death and has paved the way for personalised estimation of the risk of death in the general population. This study intends to identify the ethical resources available to approach the idea of a long-lasting dying process and consider the perspective of death prediction. The reflection on human mortality is necessary but not sufficient to face this issue. Knowledge about death anticipation in clinical contexts allows for a better understanding of it. Still, the very notion of prediction and its implications must be clarified. This study outlines in a prospective way issues that call for further investigation in the various fields concerned: ethical, psychological, medical and social.

**Methods:**

The study is based on an interdisciplinary approach, a combination of philosophy, clinical psychology, medicine, demography, biology and actuarial science.

**Results:**

The present study proposes an understanding of death prediction based on its distinction with the relationship to human mortality and death anticipation, and on the analogy with the implications of genetic testing performed in pre-symptomatic stages of a disease. It leads to the identification of a multi-layered issue, including the individual and personal relationship to death prediction, the potential medical uses of biomarkers of ageing, the social and economic implications of the latter, especially in regard to the way longevity risk is perceived.

**Conclusions:**

The present study work strives to propose a first sketch of what the implications of death prediction as such could be - from an individual, medical and social point of view. Both with anti-ageing medicine and the transhumanist quest for immortality, research on biomarkers of ageing brings back to the forefront crucial ethical matters: should we, as human beings, keep ignoring certain things, primarily the moment of our death, be it an estimation of it? If such knowledge was available, who should be informed about it and how such information should be given? Is it a knowledge that could be socially shared?

## Background

Death prediction is not a new idea, neither at the population nor individual level. Since the late seventeenth century, demographers have routinely estimated and analysed multiple population-level death-related parameters commonly summarised in life-tables. For instance, the Human Mortality Database now gathers and distributes high-quality data for 40 countries (online access: http://www.mortality.org/; consulted August 6th 2019). At the other end of the spectrum, medical teams from fields such as nephrology, intensive care and oncology take into account median life expectancy in everyday prognosis and design of treatment strategy, without always discussing this parameter with patients. Nowadays, medical doctors also use tools for very short-term predictions of death such as the Sequential Organ Failure Assessment scoring [1], about patients that are themselves most of the time too ill to actually be considering their own mortality.

In the past few years, the discovery of biomarkers of ageing has led to the development of predictors of impending natural death and has paved the way for personalised estimation of the risk of death in the general population. Recently published research has focused on the prediction of high risks of mortality in apparently healthy adults, echoing the first description in 2011 of the Smurf phenotype, a harbinger of natural death in *Drosophila melanogaster*. These recent findings suggest that the end-of-life is 1) molecularly and physiologically highly stereotyped, 2) evolutionarily conserved and 3) predictable.

Taken together, these results from independent groups studying multiple organisms including humans and using different approaches, delineate future directions in ageing research. The ability to identify and characterise individuals about to die of natural causes is a game-changer in this field. It also highlights the fact that rather than a single event, death is most fruitfully studied in the continuity of a dying process. It also brings to the forefront the notion that death is no longer only the final event of life but rather the outcome of a dying process starting much earlier with variations from one species to the other as well as amongst individuals.

There is little doubt that the discovery of predictors of impending natural death in apparently healthy adults will raise major ethical implications for both individuals and society. Such new knowledge will likely bring changes to one’s personal attitude toward one’s death. The potential public health and economic uses of this research should be investigated with equal attention.

The purpose of this study is, first of all, to clarify the meaning and scope of the ongoing research on predictors of impending natural death within the historical evolution of biological thinking about death, and within the biology of ageing. On the basis of this understanding, we will then examine the ethical resources available to approach the idea of long-lasting dying process and consider the perspective of death prediction, and we will outline issues that call for further investigation in the various fields concerned: ethical, psychological, medical and social.

## Methods

This study was based on an interdisciplinary collaboration initiated by Michael Rera, a researcher specialised in the biology of ageing, leading a project on such predictors of impending natural death, with Marie Gaille, a researcher in philosophy of medicine. Aiming for a mutual understanding and for an active debate and open conversation between colleagues, they gathered a group with a pluridisciplinary perspective: a combination of philosophy, clinical psychology, medicine, demography, biology and actuarial science. Though mostly made of colleagues working in French public research or care institutions, the group also included Katrin Solhdju (historian of sciences in the Université de Mons, Belgium, working on the issue of knowledge sharing) in one of its meetings. It resulted in a stable group (< 10 persons) and a review of the existing literature was done for each disciplinary field involved in the collaboration. The relevant literature was shared and discussed during two workshops (September 25th 2017; January 9th 2018).

In order to identify issues raised by the prospect of such indicators in the medical field, informal exploratory interviews (< 5) were made by Marie Gaille. They were qualitative semi-directive interviews (1 hr each) based on social sciences methodology [2]. They were performed with physicians and aimed at grasping how death prediction could be considered by physicians working within relatively brief time sequences (intensive care) or longer sequences of time (nephrology). In addition, several informal contacts were made with actuarians in order to understand what could be their viewpoint on such indicators.

After 1 year of exchange, it was decided to propose a collaborative and interdisciplinary paper in order, first, to highlight the specificity and novelty of the issues raised by the research on predictors of impending natural death, both from an individual and a social point of view, and second, to elaborate a combination of disciplines and viewpoints able to grasp the meaning and scope of the discovery of predictors of impending natural death and point at the need for further research on the topic.

The question was put into perspective with regard to contemporary theories of ageing as well as our understanding of what death is. Philosophy (literature review) and psychology (theory and clinical experience) have allowed us to break down into distinct themes the individual relationship to death, its anticipation and its prediction in order to better understand the challenges that the prediction of natural incoming death might raise. Our approach to the problem in the medical field has been focused on intensive care because of the high frequency of death secondary to acute illnesses. It is therefore natural that we have examined how the development of tools to predict the risk of death could become a medical decision-making tool and enable the teams involved to better cope with it. Demographic and actuarial approaches have allowed us to put prediction of death in the context of the long-standing analysis of mortality tables. Novel methods of death prediction pose new challenges to long-established assumptions of demographic models used in the implementation of pension and public health policies and insurance decisions.

## Results

### Biology of ageing: natural death as a predictable process

**Fig. 1:**
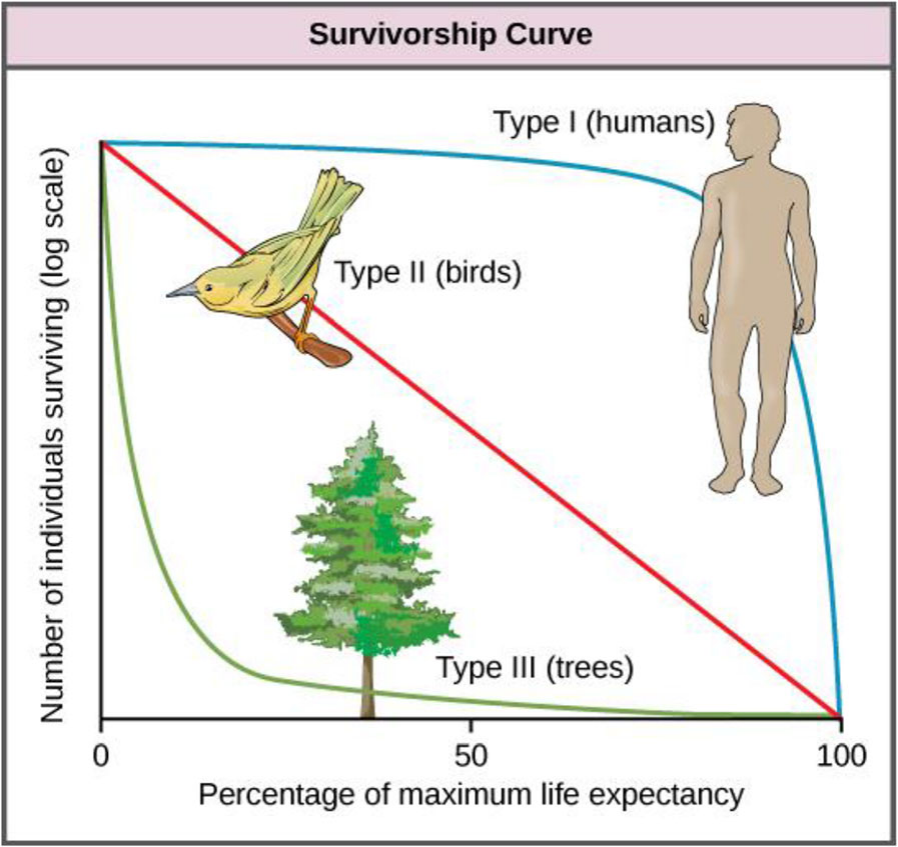
Different types of survivorship curves. Humans, most mammals and model organisms such as *Drosophila melanogaster* and *Caenorhabditis elegans* have Type I survivorship curves with death primarily occurring in old age. Birds and some reptiles have a Type II survivorship curve, whereas trees and marine invertebrates have a Type III curve. Image credit: Population demography: Figure 5 by OpenStax College, Biology, CC BY 4.0

A broad range of age-related patterns of mortality are observed across species, from negligible senescence to post-reproductive death through progressive age-dependent risk increase [3]. The present discussion mostly builds upon work conducted in *Drosophila melanogaster* and in humans. In both species increasing mortality is observed with age (the definition of “ageing” we adopt), which leads to sigmoidal survival curves or type I survivorship curves [4]. In humans, the increase in mortality with age has been acknowledged at least since the first life-table was computed by John Graunt in 1662 [5]; the exponential nature of the force of mortality for most of adult life was put forward as early as 1825 [6] (Fig. 1).

#### Early conceptions of ageing and death

Despite these long-recognised demographic regularities, the nature of ageing was persistently ignored by biologists. For decades, ageing and death were mostly seen as natural phenomena – so natural indeed that they did not warrant an explanation. Though the idea of death as a natural phenomenon was explicitly formulated by Linné, early attempts to explain death can be found in Paracelse, and later Metchnikoff, who both interpreted death as a chronic poisoning of the organism – an idea further developed by subsequent theories such as the Oxidative Stress Theory of Ageing [7].

The late nineteenth century was a turning point in the understanding of ageing. Two decades after Darwin’s Origin of Species [8] was published, German evolutionary biologist August Weisman published his own theory of ageing in mammals [9]. Its key idea was that the limitation of lifespan occurred for evolutionary reasons: since death allows the replacement and the transformation of generations, it fosters progress and evolution. The idea that ageing is a trait selected by evolution, and as such programmed, was therefore introduced.

#### Evolutionary vs. functionalist biology on death

The twentieth century staged the confrontation between two biologies – the first one seeking to answer the why (evolutionary biology); the second one the how (functionalist biology). Much greater emphasis was placed on the latter with the discovery of the structure and function of DNA, the rapid development of molecular biology techniques and, regarding ageing, the discovery of the first enzymes detoxifying the Reactive Oxygen Species [9], echoing Harman’s free radical theory of ageing [7].

On the one hand, the functionalist conception - which partly took up Weismann’s reflections - further developed the idea that there is an ageing program, coded and contained in an organism’s genome. Although Weisman explained its existence invoking evolutionary reasons (at the species level), the idea of a “programmed death” seems to go against Darwinian evolution, since fitness crucially depends on survival, i.e. postponement of death. The notion of a “program” can for instance be found in François Jacob’s work, e.g. in his book the Logic of life [10]:

“death [is] imposed from within, as a necessity prescribed from the egg onward by the genetic programme itself.”

From this perspective, death is clearly part of a process that begins, if not at birth, then at least several years before the individual’s time of death.

Evolutionary theories, on the other hand, long denied the possibility of ageing and death being deliberately programmed by the organism. They expounded on the fundamental role of chance and, in this sense, seem to be more in line with Darwinian theories.

#### Recent evolutionary theories of ageing

From the 1950s onwards, three major evolutionary theories were introduced. According to Medawar’s theory of accumulation of mutations, published in 1952, ageing is caused by the progressive accumulation of deleterious mutations with effects that manifest only late in life. Because selective pressure decreases with age (fertility being considerably higher at younger ages), such mutations are not systematically eliminated by natural selection and can be considered responsible for the development of genetic traits leading to old age and death. Williams’ antagonistic pleiotropy theory goes a little further, by inferring the existence of genes and mutations with antagonistic effects: beneficial at an early age (and therefore selected by evolution), they would also explain negative manifestations of aging and eventually lead to death. Finally, the so-called “disposable soma” theory as formulated by statistician Thomas Kirkwood, was based on the idea that the individual has a limited amount of energy, shared between reproductive functions and (non-reproductive) maintenance functions of the organism, the “soma”. According to Kirkwood’s theory, the increase in mammalian longevity is linked to a decrease in growth and reproduction rates, delaying death.

In each case, ageing was interpreted as a continuous process resulting from 1) the accumulation of deleterious mutations, 2) the expression of antagonist genes, or 3) the use of a limited amount of disposable energy – leading to an individual’s death.

#### Current functionalist approaches

These evolutionary theories coexisted until functionalist approaches became dominant in the 1980s. Evolutionary conserved mechanisms of ageing were discovered in model organisms such as *C. elegans* and *D. melanogaster* (oxidising agents, nutrients sensing deficiencies, protein aggregation, telomere attrition, cellular senescence, inflammation, etc.) [11], with the first longevity-promoting genetic mutation identified in nematodes by Cynthia Kenyon and collaborators in 1993 [12], later shown to be conserved in flies [13], mice [14] and humans [15].

Nowadays, numerous theories still coexist. Attempts have been made at classifying them into broad categories, such as: “evolutionary theories” vs. “mechanistic theories”; “programmed theories” vs. “damage/error theories”; “cellular” vs. “physiological” vs. “organ-based” vs. “genetic theories”. However, a more prevalent approach today is to try and link together the mechanisms and phenomena that are already well-known and explained. Some new theories were proposed as an attempt to build a unified version of ageing [16, 17].

#### Predictors of death

Genetically programmed or not, death may still be predicted. The most readily accessible and most widely studied predictor of the mortality risk is chronological age, i.e. the amount of time an individual has been alive. As previously mentioned, life-tables based on the mortality experience of whole populations have revealed that, in humans, the risk of death increases exponentially with chronological age for most of adult life. It should be noted that this approach yields an incomplete perspective of the ageing process at the individual level, since the interpretability of a life-table is based on the homogeneous population assumption. In cases where individuals actually experience different levels of risk, e.g. due to genetic or developmental characteristics, the population-level mortality trajectory depends on both the age-related increase in mortality and the distribution of the aforementioned characteristics [18]. Accordingly, chronological age alone fails to identify impending death situations, except at extreme ages. It has recently been estimated in Italian centenarians that the force of mortality reached a plateau at 0.645 y^−^ after age 105 [19]. In other words, approximately 50% (1-e^-0.645^) of individuals who have reached this age are expected to die within 1 year.

This line of reasoning leads to the notion of physiological age: two individuals of the same chronological age can experience different risks of impending death.

#### Death prediction based on processes

One crucial question that we have so far largely set aside is that the notion of death may refer either only to a single moment, or to a process. Indeed, death is most often represented as an event - a point on the arrow of time, e.g. in classical survival analysis methods commonly used in epidemiological or demographic research. The idea that stands in contrast to this first one is that of death as the final event of a process including a risk phase that precedes the final event, as first delineated by Buffon in his Histoire naturelle de l’homme [20].

**Fig. 2:**
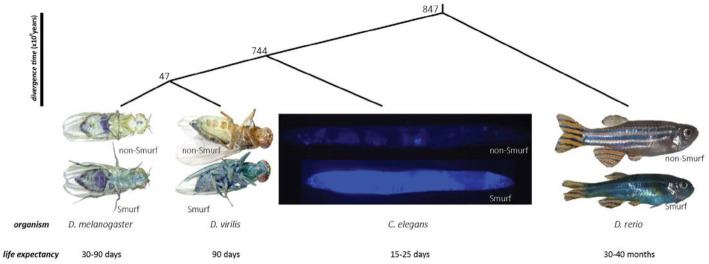
Evolutionary conservation of the Smurf phenotype through 900 million years of evolution. (published in Dambroise, E, et al. Two phases of aging separated by the Smurf transition as a public path to death. Sci. Rep. 6, (2016)

**Fig. 3:**
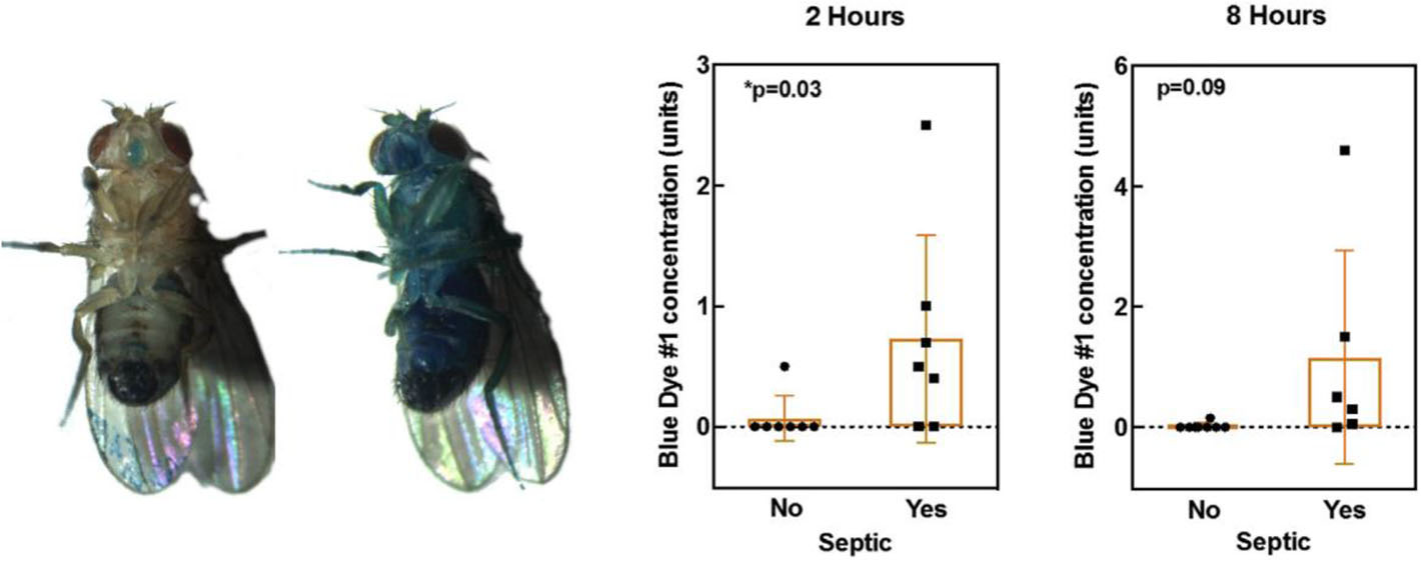
Smurf drosophila and in septic human beings. (left) picture of age-matched *Drosophila melanogaster* males that underwent the Smurf assay. In non-Smurf individuals, the blue dye is restricted to the digestive tract while in smurf individuals - showing a high risk of impending death - the blue coloration is extended to the whole body. (right) Intestinal permeability - to the blue dye #1 used in the drosophila Smurf Assay - in septic (or not) humans, 2 and 8 h after dye administration as reported in Angarita, S. A. K. et al. Quantitative Measure of Intestinal Permeability Using Blue Food Coloring. J. Surg. Res. 233, 20–25 (2019)

The latter perspective on death has been given renewed impetus by the recent identification in drosophila flies of an increase in intestinal permeability (the “Smurf phenotype”) that systematically occurs during aging and precedes impending death [21, 22]. While Smurf individuals have a markedly decreased life expectancy, compared to non-Smurf individuals, no - or little - effect of chronological age on death rates was found among Smurfs individuals. Flies randomly transition to the Smurf state, then die. Since the first description of the Smurf phenotype, in vivo assessment of intestinal barrier dysfunction associated with ageing has been performed in other model organisms [23] (Fig. 2). The interesting point to highlight here is the scaling of Smurf-phase duration depending on the life expectancy of the considered organism. From 2 to 4 days in nematodes and drosophila - in which we first described this impending death phenotype - characterized by a 20–90 days lifespan, the Smurf phase duration goes up to approximately 6 months in the zebrafish *Danio rerio*, which has a 5-year life expectancy. It is now reasonable to posit that increased intestinal permeability is linked to evolutionary conserved mechanisms and could potentially be observed in humans, although this “Smurf period” might last years [24] Results recently published by Angarita and collaborators [25] further bolster this hypothesis (Fig. 3).

This characterisation of the dying process has major implications for prediction, since observation of whether a fly has reached the Smurf state - its physiological age - predicts its risk of impending death better than its chronological age does. In humans, recent work has actually built upon the assumption that dying is a long process in the course of which progression to intermediate states can be observed and therefore yield insights into survival even for apparently healthy individuals.

Accumulating evidence supports the notion that specific biomarkers can significantly outperform more traditional risk factors and chronological age for the death prediction. Horne and coauthors for instance showed that a risk-score based on complete blood count and metabolic information led to accurate prediction of survival in the short (at 30 days) to medium term (at 5 years) in the general population [26].

In 2014, Fischer et al. performed biomarker profiling by nuclear magnetic resonance in two large groups of people [27]. They combined test results of four selected biomarkers (plasma albumin, alpha-1-acid glycoprotein, very-low-density-lipoprotein particle size and citrate) into a biomarker score that predicted all-cause mortality more accurately than any previously identified risk factors of that kind. Other research groups have shown an association between specific age-related mal- or dys-functions and mortality: for example, Pinto et al. have demonstrated that olfactory dysfunction is a strong predictor of 5-year mortality in older adults [28]. This heterogeneity amongst populations is presently modeled using frailty models and various instruments to measure biological susceptibility have also been elaborated in order to predict patient outcomes and identify individuals with a high-risk of short-term death [29–31]. Others have also focused on specific model organisms to try and elucidate significant mechanisms that are associated with ageing and short-term death [21, 32–34].

This rapidly improving ability to identify at-risk individuals, and therefore to predict with high accuracy the occurrence of death for apparently healthy individuals, evidently raises major ethical questions.

### Being mortal: philosophical and psychological approaches

The key question raised by this prospect is to determine whether tools to grasp the implications of death prediction for human beings actually do exist. We have deliberately limited the examination of these issues to human beings - which does not mean that such questioning could not be extended to other living beings. We will propose a multilevel analysis by first examining human awareness about death and then turning to philosophy and psychology, the human condition being a core subject of both.

#### Philosophy and mortality

Philosophy will be approached as an academic discipline based on a set of texts beginning with pre-Socratic fragments up to contemporary works, so as to offer a multi-layered conceptual analysis of core issues. Although both elements (i.e. the texts themselves and the conceptual analysis derived from them) vary from one cultural area to another, there are enough common features to consider philosophy here as a homogeneous and consistent body of questions and theories.

In this perspective, philosophy has paid much attention to the meaning of the mortal condition. Death is seen as, if not a defining then possibly the most elemental feature of the human condition, as illustrated in the famous syllogism “All men are mortal. Socrates is a man. Therefore, Socrates is mortal.” Formulating this point may even appear as an odd thing to do, as no one would question this fact [35].

It is often said that human beings are the only living beings who are aware of their own mortality. This awareness, however, comes with a correlated lack of knowledge: although everyone knows they are mortal, no one knows when or how they will die. This lack of knowledge is most often seen as a positive since knowing one’s own date of death has been depicted as a tragic fate. Death foretold appears tolerable only as a third-person experience [36].

This awareness can lead to the belief that human life is devoid of meaning. Although there are various attitudes towards one’s own death, including seeing it as a welcome event ([35] p. 95) to the point of committing suicide [37], human beings are generally described as facing the harshest imaginable fate: the challenge of accepting, during their lifetime, that their death is coming, and with it, the fact that their achievements, commitments or bonds will fall into oblivion, as if they had not existed [38].

Linked to this notion of human life as being meaningless, human mortality is seen as a basic impulse for philosophy itself [37] (‘On Death and its relation to the Indestructibility of our Inner Nature’, chapter XLI, Supplements to the Fourth Book, The World as Will and Idea, volume II, p. 463). As a reflection on death, philosophers have addressed the quest for immortality from a moral point of view [39].

Philosophy has mainly proposed an examination of the feelings or experience induced by the awareness of one’s future death. It has done so mostly for human beings, even though attention has been paid to animal sensitivity [40]. Twentieth century philosophy has especially focused on one feeling defined as ‘anxiety’ [41]. Such an ‘anxiety’ - or fear of death - has been commented upon since Ancient times, especially in connection with the strategies developed by human beings to escape the thought of death ([42], series IX, Diversion, 168, p. 44).

When tackling the issue of mortality, philosophers themselves have searched for ways to prevent anxiety about death: thanks to a renewed idea of death as “being nothing to us” [43], or in connection with religious consolation [42] (series XXXIV, 505, p. 122), or with a combative attitude against such an anxiety about one’s death, death being seen fully as part of human life [44] (I, XIX, p. 72; III, XIII, p. 571; I, XIX, p. 64).

#### Psychology and mortality

The analysis of psychic life has brought new insights into human awareness of death. Questioning the apparent subjective unity defended by most of Western philosophical thinking, the hypothesis of the unconscious introduces the idea that the same subject can be acted upon by multiple internal forces that do not all obey the same requirements. Psychic life would therefore be a source of conflict between conscious elements - products of logical thought in accordance with the “reality principle”, and more irrational and unconscious elements abiding by the “pleasure principle”. In this perspective, death can therefore, within the same individual, be consciously invested as rational knowledge and concurrently entirely negated by fantasies of immortality - Freud having highlighted their kinship with the workings of the unconscious mind [45].

Psychologists working with people suffering from life-threatening conditions commonly find such ambivalence in the patients’ discourse, sometimes going as far as dissociation [46]. Psychoanalyst Michel de M’Uzan radically differentiates the powerful movements that agitate the person close to death from those specific to mourning [47] associated with a peaceful acceptance of death [48]. What characterises this “work of dying”* is therefore not a form of detachment supported by a completely passive psyche. As a matter of fact, the psyche shows intense activity when approaching death: revolt, vital surge, relational appetence and even creativity of thought actually testify to the conflict at work in psychic life when facing the reality of death.

In this perspective, the emphasis is no longer on denying or accepting representations of death, but rather on the imbalance caused by their resurgence within psychic life. The psychodynamic approach therefore aims to identify the psychological processes that allow this balance to endure in order to achieve a good life, both individually and collectively; with repression being one such process. These hypotheses have been widely adopted in the fields of human and social sciences, as evidenced by many more contemporary authors who go as far as to point out a genuine collective denial of death [49]. According to them, this denial gives even more compelling force to the work of repression and, consequently, make its suppression during an actual encounter with death even less acceptable for an individual or a community. Events such as wars, epidemics, being diagnosed with a disease or a disability have an immediate effect as they shatter our “comforting illusions” [50] undermining all the processes that human societies have devised to escape the constraints nature places on each individual.

* “travail du trépas”.

### Anticipating incoming death

Philosophy and psychology have examined the meaning of death awareness for human beings, highlighting its existential and psychic implications. However, the focus on this awareness does not specifically clarify what it means to anticipate one’s own death. A specific investigation must be led on this issue.

Death anticipation is not uncommon today, particularly in health-care institutions, even though practical ways of dealing with it, both individually and collectively, are still in the process of being defined. Extraordinary situations occur where one has a precise idea of the moment of one’s death: when a person is sentenced to death, or when a person plans to commit suicide for instance.

More commonly, anticipation of one’s death occurs in “end-of-life” situations, that is to say, during a medically diagnosed time of human life - a few hours, days, weeks or even months - in which the person is considered as entering the last moments of life. We have few historical testimonies or literary descriptions of such moments (for example, in Rousseau [51], Part 6, Letter XI). Today, such situations are better documented and more numerous. This is partly due to the contemporary social organisation of death in most Western countries ([52] p.6). Another part of the explanation lies in “modern scientific capability” which has “profoundly altered the course of human life” yet not always made it possible to cure people [52]. The development of intensive care since the 1950s is paradigmatic of this “alteration”. It has led to a multiplication of cases where the patient is kept alive and conscious long enough to face the perspective of impending death. Cancerology must also be taken into account to understand this shift. In common representations, a cancer diagnosis has long meant and still often equates to some sort of death sentence [53] (p. 35). For this reason, health professionals have developed communication strategies, partial truths and step-by-step announcement of such diagnoses [54]. It has been observed that patients may both hope for a cure and be convinced that they are about to die [53]. This is still the case, even with the latest advances in cancerology, which can lead, in certain cases, to cancer becoming chronic.

Since the late 1960s, the discussion surrounding individual wishes regarding one’s own death has become widespread and reached the general public. This qualifies the aforementioned notion of both a collective and an individual denial of death. This discussion has informed the way individuals anticipate their own deaths. It is now frequent for individuals to express end-of-life preferences; manifestoes have been published in newspapers; cases have been brought to court; ethical committees have provided guidelines for health professional faced with refusals of treatment and expressed wishes to die; reforms have been passed to provide a legal framework for these new ways of facing the end of one’s life in a medical setting [55, 56].

As a result, health-care professionals, medical institutions and societies at large have begun to face the challenges posed by death-anticipation for patients. This takes the form of a vivid discussion, mostly about what medicine fails to do, could do, or should do better. First, the lack of teaching in medical curricula about the frailty of old patients, about the way they might want to live and die, and about mortality in itself is criticised ([52] p.9). The necessity to make up for it in contemporary health-care systems is now emphasised. In addition, the development of palliative care in health-care systems over several decades is viewed as a way to provide answers for this issue, though it has also been criticised for conveying questionable conceptions of what constitutes a “good death” and a “good way” to face one’s death [57]. For several decades, certain health-care institutions have integrated a new way of practising medicine, no longer focused on trying to cure, but rather on providing care to dying persons. Finally, palliative care is not the only answer to criticism aimed at current medical education. Death-anticipation gives rise to other types of initiatives such as the recent “anticipated discussions”, which give patients the possibility to discuss their “end-of-life” with their physicians [58].

Advance directives and care for people at the end of their lives stand in contrast to a medicine that states, in the face of therapeutic failure, that “there is nothing more to do”. On the contrary, the palliative care movement, and more broadly the philosophy of care, are based on the idea that “there is always something to do with a patient” provided that the care process is focused on the person’s needs and wishes regardless of curative possibilities [59].

Psychology actually tells us that it is crucial to maintain one’s creativity and ability to think and feel emotions in extreme and unpredictable situations. This necessity can be seen in relation to the infant’s state of ontological precariousness, with the ability to emerge from it depending on the quality of the emotional and human environment provided (Vol. 1) [45, 60, 61]. Freudian and post-Freudian research has carefully examined how, for infants, an experience of extreme helplessness in response to an original state of distress becomes the starting point of a trauma [60]. Traumatic feeling comes with “a high state of passivity”, i.e. a constrained and unwanted experience closely related to this original state of distress. As such, only a permanent work of anticipation based on a reliable social environment may allow the individuals concerned to protect themselves from it. Experiences in clinical psychology in the context of degenerative diseases, certain types of cancer or AIDS - in cases where therapeutic impotence had not been overcome – show that people may be unable to modify or reduce this vulnerability for a period of time, which inevitably results in a traumatic experience [62]. Previous research and authors have shown that the quality of human support and the environment reduces traumatic impact and, even more so, post-traumatic distress. Giving space to the patient’s wishes thus alleviates their feelings of powerlessness and helps them cope with their situation.

Moreover, human creativity, which is so important in human life, would then be considered by some other authors as the key element of the work of ageing [63]. Death appears as a consequence of the impossibility to continue to age: that is, to keep on transforming somatic and psychic processes in order to maintain the balance between the various requirements that allow the continuation of life. The reliability of human beings stems from their ability to survive in conditions where their greatest precariousness is revealed.

In both this section and the previous one, the relationship to one’s own death is based on the notion of death as an event interrupting life. Death is considered as the final term, just as it is when used in its metaphorical sense^77^. Anticipation of death is not uncommon but, so far, it has been considered when taking place in a medical context where the person in care suffers from a lethal disease or very poor health condition due to old age. However, the research into predictors for impending natural death implies that death prediction could become relevant to anyone, at any age, and not necessarily solely to persons suffering from a condition. This notion has been sketched out once before, but not really developed so far ([51, 52] p. 75). As a result, we must now take another step in our reflection and consider the specifics of death prediction as understood within the research on predictors of impending natural death.

## Discussion

### Individual implications of mortality prediction

If such predictors were to become widely available, anyone could experience entering a phase of life set to a “countdown” (which could last several years). This phase could be described as a very different dying process from what is today labelled as “end-of-life”, and from the experience described by anthropologist Todd Meyers about “Beverly”, a person with multiple chronic conditions, who refers to her own life as one long dying process [64]^78^. In addition, the notion of prediction of death may differ from that of anticipation of death.

In the context of this possible future, we address some implications of the widespread availability of tools of this kind. In this last section of the study, we propose a first sketch of the implications of predicting death as such, which differs from the awareness of death or even of the anticipation of death as approached in the two previous sections. We will pay special attention to the process as experienced by the person informed of such a prediction, it’s possible uses in medical decision-taking, and finally its possible social and economical uses. We thus hope to anticipate the potential outcomes of contemporary biological research on ageing and identify directions for further investigation in that field.

Though still largely theoretical, the experience of entering a phase of life set to a “countdown” probably has some common features with a situation we are familiar with: namely, the situation arising from genetic testing made in pre-symptomatic stages of a disease. Let us recall here the main points of ethical analysis developed in such a situation. It may help us to foresee the implications of death prediction itself. Particularly interesting insights may be derived from the case of pre-symptomatic testing for Huntington’s disease. Reflecting on a family history in her book Mapping Fate: A Memoir of Family, Risk, and Genetic Research, Alice Wexler recounts the life of the author’s mother whose health slowly deteriorates because of Huntington’s disease, and the action initiated by her sister and her father to discover the gene causing the disease [65]. The use of the word “fate” suggests that, after receiving their test results, the person affected will not see their life as an open future, but as a timespan closing in on itself.

This finite course of time appears to be the main issue at stake [66]; a point that echoes Vladimir Jankélévitch’s analysis of death: on the basis of Victor Hugo’s work of fiction, The last day of a condemned man, he emphasised that a death sentence leads a person to enter “an unbearable and inhuman” time, where one’s time of death is known [67]. Nietzsche’s anthropological perspective - according to which it is better for human beings not to know their future - appears as strikingly relevant on this matter [68]. Along the same lines, Jean-Claude Ameisen states that we live in a state of constant forgetfulness of our previous metamorphoses, letting the memory of all the ones that occurred before sink into oblivion [69]. In other words, we require a certain ignorance of the future in order to be able to live. Having one’s forthcoming death announced completely breaks with this dynamic and, as such, any framework for disclosure should take into account the time and support needed to recalibrate one’s choice between ignorance and awareness [70].

Ethical discussions on genetic screening, be it for adults (as used to detect risks of breast cancer) or for children (as used to detect risks of cystic fibrosis or neuromuscular diseases) tend to reach similar conclusions [71–74]. As a result, undergoing genetic testing is not an obvious decision to make. Interestingly, only a minority of people who do make it actually complete the process [75]. This information alters the individual’s health status yet it does not provide them with any medical response or treatment. In doing so it may definitely raise levels of stress and impair possibilities of planning the future. It also raises the question of the status of the individual who tests positive: will they be considered a patient of medicine, especially so if the announcement comes from a medical actor? How can this announcement be managed whilst patients are totally asymptomatic and do not feel sick or near death? What kind of support can be offered to them and should it be strictly medical?

These unresolved questions clearly show specific attention should be paid to the ethical implications of death prediction. In terms of decision-making, they call for the elaboration of a special process, probably unfolded over a long time period, and on several stages.

### Medical applications and related issues

Being able to precisely predict impending death would inevitably have medical implications. We shall give a few examples of those potential implications and discuss ensuing issues. We will focus first on the case of an acutely ill patient with a so-called fatal prognosis, then on a healthy subject diagnosed with a fatal disease. Scientific progress over the past century has considerably increased our understanding of human physiology, but many aspects of it are still unknown. Death prediction is naturally dependent on the progress of research and medical advances, but it is a new parameter in our end-of life appraisal.

#### Acutely ill with fatal prognosis

After traumatic brain injury, cardiac arrest or septic shock, patients may suddenly become comatose or suffer multiple organ failure requiring organ support. Treatment for such diseases is dispensed in intensive care units (ICU) and can last up to several weeks at a time. Both patients and family are always very affected by an ICU hospitalisation and relatives may often want to know the prognosis of the disease. It is unlikely that anyone would wish to carry on with invasive treatments in the ICU for a patient with a 100% probability of short-term death. For the most severe cases, clinical scores based on multiple exams - clinical, radiological and biological - are precise enough to provide guidance as to the withdrawal of treatment, which is a legal procedure in France [76–78] and simply a recommendation in the UK [79]. For comatose patients after cardiac arrest, some factors indicating very poor outcome are also now identified [80]. But for all other patients, their precision remains low (with a C-statistic value of 0.82 at best) [81].

In such cases, having a robust predictor of fatal outcome would be very helpful. It would help shorten the length of stay in the ICU, avoid pointless suffering for the patient and allow more time for next-of-kin support [82, 83]. It would also make it possible to better allocate resources between therapeutic care for patients who are more likely to survive and palliative care for those with a very reliable predictor of impending death. But to be implemented within the routine of care, such a predictor will have to rely on large cohorts as well as on a robust pathophysiology. It means that increasing the number of cases in the database will not be enough, due to the high-variability of human bodies and conditions. In the modern world of machine learning and “artificial intelligence”, such a score will have to remain relatively low on the vertical line of “The Axes of Machine Learning and Big Data” yet remain consistently as high as possible on the horizontal line [84]. Numerous initiatives are now expanding on these issues [78, 85].

#### Patient fit and well-being diagnosed

Thanks to recent advances, a previously healthy person diagnosed with a fatal disease may be given a reasonable idea of their remaining life expectancy. From a medical perspective, even in the absence of a known cure, an optimistic approach may consist in trying everything to increase the life expectancy of the patient, including active therapy but also avoidance of treatment inasmuch as it may exacerbate the underlying condition. From a research perspective, studying the way of life of the patient and other comorbidities will help practitioners understand why a certain pathology may worsen and how to prevent it.

From a pragmatic point of view, knowing that a patient is due to die (i.e. in the coming months) will raise ethical problems regarding which treatments to offer this patient. For example, a patient diagnosed with a fatal disease and who suffers acute kidney injury or metastatic disease may not necessarily benefit from long-term dialysis or chemotherapy. Within the medical community this type of discussion is already commonplace, if not a daily occurrence, for a broad range of patients, yet it is sometimes addressed with imprecise tools. Indeed, medical progress has made many pathologies survivable and there is nothing unusual today in treating an 80-year-old patient for his second or third cancer. Adding an objective tool will help medical teams devise the best plan of care in accordance with the patient’s wishes.

But again, it is important to emphasise here that the prediction of life expectancy may remain indicative for a long time, to be taken into account among several others rather than as a binary answer. In addition, it remains to be investigated from an ethical point of view what the use of such a parameter could imply within a patient-based approach of medical decision.

### Implications for society: the case of longevity risk

The development of predictors for impending natural death would also change the way longevity risk is perceived, both from a social and an individual point of view. The availability of accurate death predictors could dramatically change how the risk of outliving one’s savings is perceived by individuals. As a consequence, the design of state pensions and long-term care systems could be modified.

Managing social and economic challenges produced by the unpredictable nature and sustained improvement in human lifespan is an age-old issue. As discussed in the introduction, the first age-specific mortality model [6], motivated by the pricing of life annuities [86] was published in 1825. Since then, there has been a spectacular development of mortality models (see e.g. [87–90]), used for evaluating pensions and public health reforms or regulatory reserve for pension funds and insurance companies [91].

This so-called Longevity risk is usually subdivided into several components, including a systematic mortality risk - arising from unknown future improvement in mortality reduction; and an idiosyncratic risk - correlated to the unpredictability of an individual’s date of death.

First, personalized measurement of physiological age would substantially reduce the systematic risk. Such measures would open a whole new field of research for developing more accurate mortality models, which could be based on observable frailty indicators, and thus a better assessment of the longevity risk for society.

Pension and long-term care providers (states, pension funds, insurance companies …) cannot evaluate how long benefits will need to be paid to an individual and are therefore faced with significant idiosyncratic risk in addition to the systemic risk. Having access to accurate individual death predictors could reduce this idiosyncratic risk by reducing the residual variance of late-life phase individuals. This would be particularly relevant for small providers, heterogeneous groups or individualised retirement systems in which individuals bear their own longevity risk.

But the implementation of death predictors is also likely to challenge solidarity and risk-pooling principles on which many retirement and long term-care systems are built, thereby encouraging more individualised organisations. Indeed, the unpredictable nature of one’s date of death is a strong incentive for one’s willingness to contribute to the pool and risk-sharing systems, which entail both the prospect of obtaining less than contributed (for instance in retirement benefits) and the counterbalancing insurance against one’s own longevity risk (risk of outliving one’s savings). However, if better death predictors were to become available, should individuals with a shorter remaining lifespan be authorized to withdraw from retirement systems that share the longevity risk?

This looming issue is reminiscent of the current questions surrounding solidarity between groups known to have different life expectancies. For instance, it is already the case that insurance companies are not allowed to charge higher premiums to women [92], including for longevity related products, even though women are known to live longer than men. Some concerns have also been raised regarding “unfair” redistribution properties of public pension systems in the presence of socioeconomic differences in mortality (see e.g. [93–96] the determinants of mortality). Indeed, these systems could produce “an undesirable transfer of wealth away from lower socioeconomic groups with shorter life expectancy to higher socioeconomic groups with above average longevity” [94]. The magnitude of such challenges would be vastly expanded in the presence of individualised death predictors. The solidarity principles of pensions and long-term care systems could be jeopardised, or at least redefined.

The most urgent question to answer might be: which institutions, if any, should have access to information on death predictors? For instance, should an individual have to disclose the information that they have entered the “countdown” phase of their life? Again, this issue echoes present ones, since policyholders already have to disclose to insurance companies pieces of information (smoking habits, previous illnesses …) known to be determinants of mortality [97] Thus, the availability of massive amounts of individual data relevant for death prediction might be one of the most important regulatory challenges of the coming decades.

The literature on the governance and ethics of genetic information may be helpful to consider this issue as there are some analogies between death predictors and personal genetic data, the individual’s “intimate diary” according to the phrase coined by G. Annas [98]. For two decades, this literature has explored various ethical and political models that could “accommodate the connected nature of the genetic self”, if not protect confidentiality (such as the benefit sharing model or the trust model [99]); it also calls for a new social contract [100]. It is interesting to observe the debate on the need for a constitutionally-entrenched right to privacy presently taking place in the US, as the various existing regulations, especially the Health Insurance Portability and Accountability Act/HIPAA (2000) and the Genetic Information Non-Discrimination Act/GINA (2008), apply only to a minority of citizens, not to for-profit companies such as GEDmatch, 23andMe, or Ancestry.com; nor to the “All of US” Program, its private partners, nor to websites or apps.

## Conclusions

The biological research on ageing presented in the first part drove us to investigate the ethical, medical and social implications of death prediction both from an individual and a collective standpoint. Based on a multidisciplinary approach specifically focused on biology, philosophy, psychology, medicine, demography and actuarial sciences, we argue the necessity to distinguish the awareness of one’s own mortality, the challenge of anticipating one’s death, and finally the issues that arise from predicting it. We also identify multiple forthcoming issues, such as conditions and consequences of a (potential) clinical use of death prediction, risks associated with private interests taking control of information obtained in that context, and potential increased psychological vulnerability.

These issues should be addressed upstream to any application of the increasingly-available tools for assessing individual remaining lifespans. They raise multiple challenges while offering promising opportunities, especially to determine the proper choice between palliative and curative approaches. To fully grasp them, the investigation must continue in relations with the questions we outlined in this work: What kind of medicine and health-care do we want? What collective organisation do we want to adopt, in terms of insurance? What are we, as human beings, able to apprehend and what would we prefer to ignore? What information about ourselves do we allow to circulate and do we accept to share?

## Data Availability

Not applicable.
